# Enterovirus Co-infections and Onychomadesis after Hand, Foot, and Mouth Disease, Spain, 2008

**DOI:** 10.3201/eid1712.110395

**Published:** 2011-12

**Authors:** Maria A. Bracho, Fernando González-Candelas, Ana Valero, Juan Córdoba, Antonio Salazar

**Affiliations:** Centro Superior de Investigación en Salud Pública, Valencia, Spain (M.A. Bracho);; Centro de Investigación Biomédica en Red en Epidemiología y Salud Pública, Barcelona, Spain (M.A. Bracho, F. González-Candelas);; Universitat de València, Valencia (F. González-Candelas);; University of Edinburgh, Edinburgh, Scotland, UK (A. Valero);; Hospital Universitario La Fe, Valencia (J. Córdoba);; Centre Salut Pública de València, Valencia (A. Salazar)

**Keywords:** enterovirus, hand, foot, and mouth disease, HFMD, onychomadesis, co-infection, viruses, Spain, nail shedding, complications

## Abstract

Hand, foot, and mouth disease (HFMD), a common disease caused by enteroviruses (EVs), usually affects children. Clustered and sporadic HFMD cases, followed by onychomadesis (nail shedding), occurred during summer and fall 2008 in Valencia, Spain. Fecal samples from onychomadesis patients, who did or did not have previous HFMD, and from healthy children exposed to onychomadesis patients tested positive for EV. The complete viral protein 1 capsid gene sequence was obtained for typing and phylogenetic analysis. Two EV serotypes, coxsackievirus A10 and coxsackievirus B1 (CVB1), were mainly detected as a monoinfection or co-infection in a childcare center where an onychomadesis outbreak occurred. On the basis of our results, and detection of CVB1 in 2 other contemporary onychomadesis outbreaks in childcare centers in Spain, we propose that mixed infection of an EV serotype that causes HFMD, plus the serotype CVB1, could explain the emergence after HFMD of onychomadesis, a rare and late complication.

## Abstract 2

Mixed infection of enteroviruses may explain the rare complication of nail shedding.

Onychomadesis after HFMD

Enteroviruses (EVs) are among the most common human viruses, infecting ≈1 billion persons worldwide annually (*1*). On the basis of phylogenetic analysis, the genus *Enterovirus* (family *Picornaviridae*) is divided into 10 species. Members (serotypes) of human enteroviruses (HEVs) are classified into 4 species: HEV-A, HEV-B, HEV-C, and HEV-D (*2*). Although most EV infections are asymptomatic, they can result in a broad range of clinical manifestations, ranging from benign symptoms to notable diseases such as poliomyelitis, severe neonatal systemic disease, encephalitis, meningitis, or myocarditis (*3*).

Hand, foot, and mouth disease (HFMD) typically affects children <10 years of age. The main signs and symptoms are fever; sore throat; general malaise; and, often, vesicular eruptions on the palms of the hands, soles of the feet, oral mucosa, and tongue. Although HFMD is classically a mild disease, outbreaks in Asia have been associated with a high incidence of fatal cardiopulmonary and neurologic complications (*4*). EVs that are most frequently reported as causing HFMD outbreaks include EV71 and coxsackievirus A16 (CVA16) (*5*). Other HEV-A serotypes, such as CVA4, CVA5, CVA6, and CVA10, have also been reported in cases of HFMD and herpangina, a disease that shares clinical symptoms with HFMD (*6**–**9*). HFMD, followed by onychomadesis (nail shedding), was first reported in 2000 in 5 children in Chicago, Illinois, USA (*10*). In 2001, a similar report described it in 4 children in Europe (*11*). Since 2008, several onychomadesis outbreaks (HFMD outbreaks followed by onychomadesis) have been reported in various locations in Spain: Valencia (*12*), Valladolid (*13*), Saragossa (*14*), and A Coruña (*15*). A preliminary case–control study from the 2008 Valencia onychomadesis outbreak established a clear link between HFMD and onychomadesis (odds ratio 5.836, p<0.001) (*12*). Finally, onychomadesis cases in the context of a HFMD outbreak have also been reported in Finland in 2008 (*7**,**16*).

Molecular characterization of the etiologic agent involved in onychomadesis after HFMD, either in clustered or sporadic cases, remains controversial. Although serotypes CVA6 and CVA10 co-circulated during the 2008 HFMD outbreak in Finland (*16*), only CVA6 was explicitly reported in the HFMD cases in which the patients experienced onychomadesis (*7*). In Spain, serotype CVB1 was detected in A Coruña, and both CVB1 and CVB2 were detected in Saragossa. Noticeably, serotypes CVA10 and CVB1 were prevalent in the preliminary reports of the 2008 onychomadesis outbreak in Valencia (*17*). Considering all of these results together, no convincing demonstration has been made to clarify which serotype could account for the HFMD–onychomadesis epidemics.

In this study, to establish a relationship between EV infection and the onychomadesis patients in Valencia, Spain, in 2008, we analyzed fecal specimens from children who experienced onychomadesis after HFMD and from healthy children who had been in contact with onychomadesis case-patients. As a result of identifying EV serotypes and conducting phylogenetic analyses of viral protein (VP) 1 gene sequences, we propose that either co-infection or superinfection with an EV serotype that causes HFMD, along with serotype CVB1, could explain the emergence of recent HFMD–onychomadesis outbreaks. However, further research on future onychomadesis outbreaks that overcome the limitations of this study are necessary to verify this proposal.

## Materials and Methods

### Patients and Clinical Samples

Fecal samples were obtained from children in clusters of cases or from children with sporadic onychomadesis cases and from their asymptomatic classmates or contacts who were exposed to onychomadesis patients. All study participants were identified during May–December 2008 in Valencia. An onychomadesis case-patient was defined as a person who had lost >2 fingernails or toenails unrelated to systemic disease or trauma.

### Viral RNA Purification, Reverse Transcription PCR, and Sequencing

Viral RNA was purified from feces by using Nuclisens EasyMag automated extractor (bioMérieux, Durham, NC, USA). Samples that rendered an enterovirus-positive result after real-time amplification (Cepheid’s Xpert EV, Sunnyvale, CA, USA) were selected for typing. Sequences corresponding to the gene encoding the entire VP1 protein were obtained by reverse transcription PCR (RT-PCR) followed by direct sequencing. Most of the amplicons corresponding to either HEV-A or HEV-B serotypes were generated by using generic primers 011, 055, 224, and 240 (*18*). cDNA was synthesized in a 20-µL volume reaction containing 10 µL RNA, 500 µmol/L dNTP, 100 U Moloney murine leukemia virus reverse transcriptase (Promega Corp., Madison, WI, USA), 20 U RNasin (Promega) and 1 µmol/L antigenomic primer 240. The RT mixture was incubated at 42°C for 60 min, followed by 4 min at 95°C. A first PCR was performed in 50 µL volume containing 5 µL cDNA, 5 µL 10× PCR buffer, 0.2 mmol/L of each dNTP, 0.4 µmol/L primer 224, 0.4 µmol/L primer 240 and 1.25 U recombinant *Taq* DNA polymerase (TaKaRa Bio Europe SAS, Saint-Germain-en-Laye, France). A nested PCR was subsequently performed in a 50-µL volume with primer pair 011/055. PCR profiles were 94°C for 2 min; 40 cycles at 94°C for 30 s, 50°C for 30 s, and 72°C for 3 min; and a final extension step at 72°C for 10 min.

The above-described generic RT-PCR strategy was complemented with an additional species-specific strategy. The species-specific protocol was performed for all samples and consisted of using the same RT-PCR conditions but with the specific primers for HEV-A and HEV-B as described (*19*). In all cases, the region encompassing the entire VP1 gene was amplified in 2 overlapping fragments. cDNA was synthesized with either antigenomic primer 489 (HEV-A) or 493 (HEV-B). First-round PCRs included outer pairs 486/489 (HEV-A) or 490/493 (HEV-B); in the second round, 2 heminested PCRs were performed with either pairs 486/488 and 487/489 (HEV-A) or pairs 490/492 and 491/493 (HEV-B). When necessary, for mixed infection, additional PCRs were performed with additional primers (Table 1). Purification of amplicons and sequencing was performed as described (*20*). All primers mentioned were considered potentially useful for PCR or sequencing in both generic and specific strategies. GenBank accession numbers for sequences derived in this study are FR796476–FR796493 and FR797984–FR798004.

**Table 1:** Primers designed in this study used to amplify and sequence the VP1 gene region*

Name	Sequence, 5′ → 3′	Gene	Virus	Sense	Position	Use
292a	CACCNGTYTCIRCIGC	VP1	EV	A	2582–2597	Seq
7g	TGCTGCARTATATGTATGT	VP1	EV-A	G	2879–2897	Amp, seq
EV71vp1g2	ATGTTTGTACCACCCGGAGCCCC	VP1	EV71	G	2894–2916	Amp, seq
CA10vp1g2	ATGTATGTGCCCCCTGGCGCCCC	VP1	CVA10	G	2894–2916	Amp, seq
CA10_55	GGGACGCATGTGGTGTGGGA	VP3	CVA10	G	2162–2181	Amp, seq
CA10_11	GCGCCGGATTGGTGGCCAAA	A2	CVA10	A	3326–3345	Amp, seq
vp1CA10 g1	TRCAGGCTGCAGAGACGGG	VP1	CVA10	G	2567–2585	Seq
vp1CA10a1	GATGGGTTAGTTGCTGTTTGCCA	VP1	CVA10	A	2945–2967	Seq
vp1CA10a2	GGGGGCACATACATATATTG	VP1	CVA10	A	2888–2907	Seq

*Primer positions are relative to the CVA2-Fleetwood sequence (GenBank accession no. AY421760). VP, viral protein; EV, enterovirus; A, antigenomic; seq, sequencing; G, genomic; amp, PCR amplification; CV, coxsackievirus.

### Sequence and Phylogenetic Analysis

HEVs were genotyped by sequence comparison by using BLAST (http://blast.ncbi.nlm.nih.gov/Blast.cgi). Alignments of 5′ and 3′ partial VP1 sequences were obtained with ClustalW (*21*). Maximum-likelihood phylogenetic trees were constructed by using RAxML version 7.2.6 (*22**,**23*) with the general time-reversible model of nucleotide substitution, a gamma-distribution approximation to account for rate heterogeneity and bootstrap support for branches by using 1,000 replicates. Trees were edited with the Tree Explorer tool in MEGA4 (*24*).

## Results

### Studied Persons

Sixty-five fecal samples, collected from 44 onychomadesis case-patients (28 with HFMD) and 21 children who were exposed to onychomadesis case-patients (3 with HFMD), were tested for EVs. Of these, 38 (59%) samples collected from 29 onychomadesis case-patients (23 with HFMD) and 9 exposed persons (1 with HFMD) tested positive. To eliminate likely incidental serotypes not related to onychomadesis, we selected clinical samples from children with sporadic onychomadesis–HFMD by using the following tentative exclusion criteria: time between HFMD symptom onset and specimen collection >90 days and a latency period between the onset for HFMD and the onset for onychomadesis of <2 weeks, which is considerably shorter than the corresponding average in other onychomadesis outbreaks (≈40 days). As a result, 6 EV-positive samples were excluded from the study.

All 32 children studied (19 boys) were <5 years of age (mean 2.1, range 1.3–4.2 years) (Table 2). They attended 9 different childcare centers, except for 5 children, 3 of whom were siblings. A cluster of onychomadesis cases during May–July 2008 was first reported in childcare center 1 from which 17 samples from onychomadesis patients and exposed children were studied. By the time the epidemiologic study was conducted in childcare center 1, some sporadic onychomadesis cases were reported in other childcare centers, and EV-positive samples from corresponding case-patients and 1 exposed person were analyzed (childcare centers 2–8). In October 2008, another cluster of HFMD cases was identified in childcare center 9, from which fecal samples were taken even before onychomadesis symptoms developed in HFMD case-patients. In our study, nail shedding appeared an average of 32 days after HFMD onset (95% confidence interval [CI] 24–39 days), and fecal samples were collected an average of 44 days after HFMD onset (95% CI 35–54 days) and 25 days after onychomadesis onset (95% CI 18–32 days), excluding data from samples collected before onychomadesis onset (negative days in Table 2).

**Table 2:** Clinical and epidemiologic data for symptomatic and asymptomatic children and genotyping results of 32 fecal samples collected in the HFMD–onychomadesis outbreak, Valencia, Spain, 2008*

Childcare center	Isolate no.	Age, y/sex	HFMD	Date of onset for HFMD	Onych	Date of onset for onych	Date of sampling	Serotypes	Days from HFMD onset to sampling	Days from onych onset to sampling
None	54574	1.5/F	Yes	Apr 27	Yes	May 27	Jun 25	CVA10	58	28
	54696	1.7/F	Yes	May 24	Yes	Jun 13	Jul 1	CVA10	37	18
	54697	1.7/F	Yes	May 24	Yes	Jun 13	Jul 1	CVA10	37	18
	54698	1.7/M	Yes	Jun 24	Yes	Jun 13	Jul 7	CVA10/CVA6	37	18
	54682	1.4/F	Yes	Jun 9	Yes	Jun 23	Jun 30	CVA16	21	7
1	54624	2.4/M	Yes	May 9	Yes	Jun15	Jun 26	CVA10	47	41
	54628	2.3/F	Yes	May 3	Yes	May 18	Jun 26	CVA10/E9	53	38
	54629	1.7/M	Yes	Apr 18	Yes	Jun 28	Jun 26	CVB1	68	28
	56643	1.8/M	Yes	May 1	Yes	Jun 19	Jun 26	CVA10	55	37
	54582	1.5/M	Yes	Apr 20	Yes	Jun 20	Jun 25	CVA10/CVB1	65	5
	54636	1.3/M	Yes	May 12	Yes	Jul 7	Jun 25	CVA10/CVB1	43	–12
	54602	2.2/F	Yes	Apr 15	Yes	Jun 9	Jun 25	CVA10/CVA5	70	16
	54573	1.4/M	No		Yes	May 2	Jun 25	CVB1		50
	54576	2.0/M	No		Yes	May 30	Jun 25	CVA10		25
	54622	2.4/M	No		Yes	Jun 3	Jun 26	CVA10		23
	54667	1.4/M	No		No		Jun 29	CVA10/CVB1		
	54572	2.4/M	No		No		Jun 25	CVA10		
	54575	3.2/F	No		No		Jun 25	CVA10		
	54579	1.4/M	No		No		Jun 25	CVB1		
	54601	2.0/M	No		No		Jun 25	CVA10		
	54599	2.9/M	No		No		Jun 25	CVA10		
	54632	3.4/M	No		No		Jun 27	CVB1		
2	54693	2.8/M	Yes	May 21	Yes	Jun 25	Jul 1	CVB1	40	6
3	54657	2.1/F	Yes	May 13	Yes	Jun 21	Jun 26	CVA6/CVB1	43	5
4	54753	4.2/M	No		No		Jul 2	CVA10		
5	54694	1.6/F	Yes	May 20	Yes	Jun 5	Jul 1	CVA6	41	26
6	54633	2.8/M	Yes	NA	Yes	May 15	Jun 27	CVA5		
7	54678	1.7/F	Yes	Apr 1	Yes	May 14	Jul 1	EV71/CVA5	90	47
8	54720	1.7/F	No		Yes	May 1	Jul 2	EV71		61
9	1023	1.9/F	Yes	Oct 18	Yes	Nov 28	Nov 4	CVA6	16	–31
	1215	2.0/F	Yes	Oct 21	Yes	Dec 1	Oct 31	CVA6	9	–24
	1031	2.0/M	Yes	Oct 23	No		Nov 4	CVA6	11	

*HFMD, hand, foot, and mouth disease; onych, onychomadesis; none, no childcare center attendance; CV, coxsackievirus; E, echovirus; NA, not available; EV, enterovirus.

### EV Typing

All EV-positive samples could be typed, and 7 different serotypes were found, 5 belonging to HEV-A species (CVA5, CVA6, CVA10, CVA16, and EV71) and 2 to HEV-B (CVB1 and echovirus [E] 9). In 4 samples, CVB1 was found in dual infections with either CVA10 or CVA6. The most prevalent serotypes were CVA10 (45%) and CVB1 (22.5%), which were mainly detected in childcare center 1 and in children not attending any childcare center, followed by CVA6 (15%), the only serotype detected in the 3 children from childcare center 9. Other serotypes were found with more marginal frequencies: CVA5 (7.5%), EV71 (5%), CVA16 (2.5%), and E9 (2.5%). Sporadic onychomadesis cases that matched exclusion criteria for likely incidental infections were analyzed and presented HEV-B serotypes CVB3 (n = 2), E3 (n = 1), E9 (n = 2), and E3/E9 co-infection (n = 1). A substantial number of mixed infections (25%) was detected. All 3 mixed infections CVB1/CVA10 occurred in childcare center 1 and were found in 2 symptomatic children (with both HFMD and onychomadesis) and 1 healthy child. Thus, mixed infection of both serotypes was found in children who stayed in the childcare center where the first onychomadesis case were identified. Other combinations of mixed infections were detected only once.

Additionally, viral extracts from 16 EV-positive samples were inoculated into cell culture (human cervical carcinoma, human rhabdomyosarcoma, and human embryo fibroblasts), followed by EV detection with immunofluorescence (data not shown). Eight samples typed as HEV-A (mixed or monoinfection) produced negative results, whereas all 5 samples typed as CVB1 and all 3 samples with the mixed infection CVA10/CVB1 showed positive results.

### Phylogenetic Analyses

**Figure 1:**
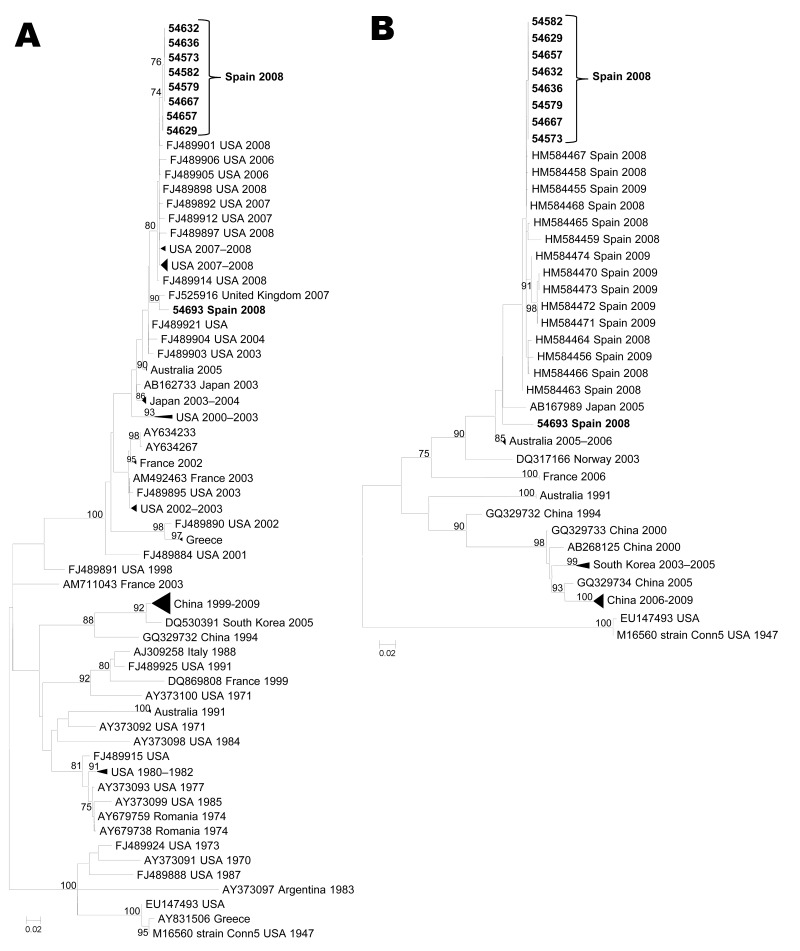
Maximum-likelihood phylogenetic reconstructions for coxsackievirus B1 based on partial viral protein 1 sequences. A) 5′ partial coding region (93 sequences, 294 nt; B) 3′ partial coding region (49 sequences, 390 nt). Bootstrap values >75% are shown. Scale bars indicate number of substitutions per nucleotide position. Multiple strains from the same country sharing the same node were collapsed and shown as triangles with shape proportional to branch distances and number of sequences.

**Figure 2:**
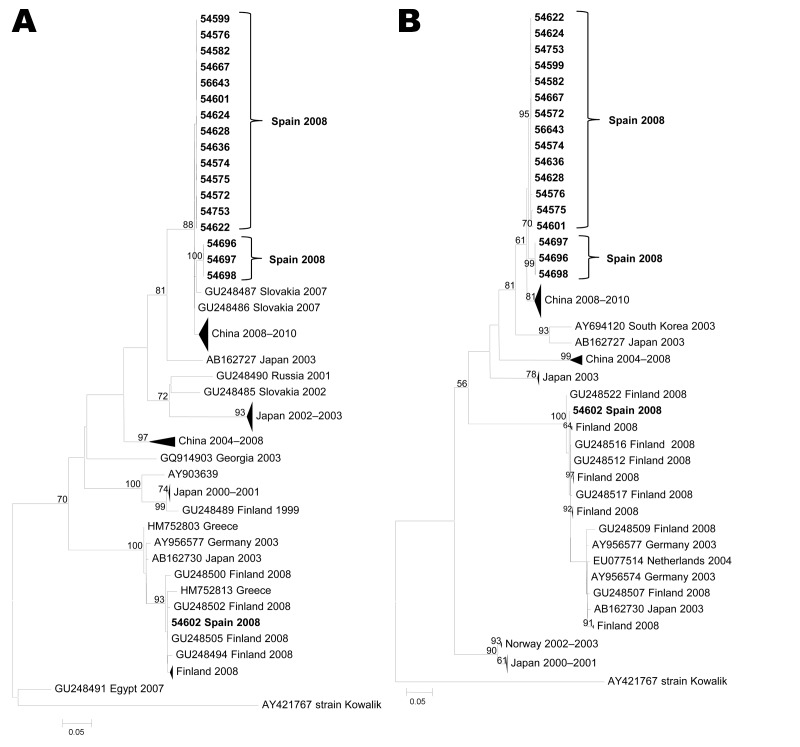
Maximum-likelihood phylogenetic reconstructions for coxsackievirus A10 based on partial viral protein 1 sequences. A) 5′ partial coding region (89 sequences, 246 nt); B) 3′ partial coding region (87 sequences, 397 nt). Bootstrap values >75% are shown. Scale bars indicate number of substitutions per nucleotide position. Multiple strains from the same country sharing the same node were collapsed and shown as triangles with shape proportional to branch distances and number of sequences.

**Figure 3:**
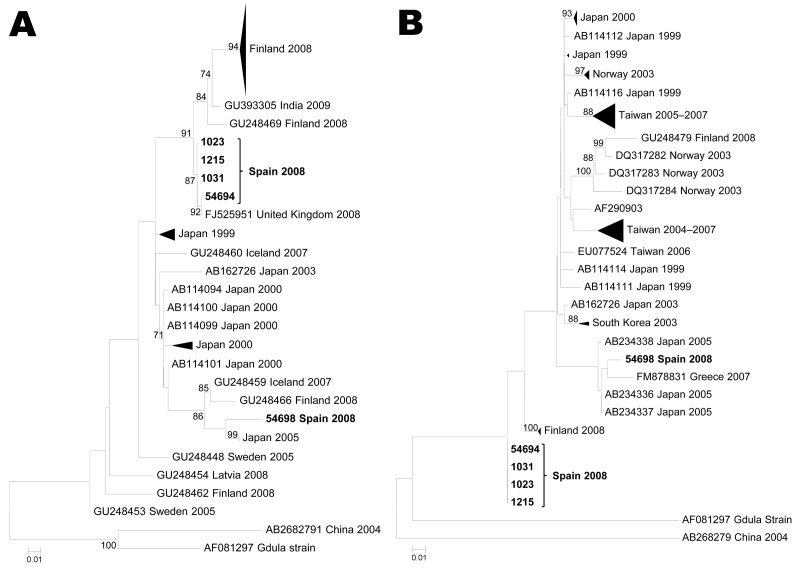
Maximum likelihood phylogenetic reconstructions for coxsackievirus A6 based on partial viral protein 1 sequences. A) 5′ partial coding region (81 strains, 293 nt). B) 3′ partial coding region (68 sequences, 377 nt). Bootstrap values >75% are shown. Scale bars indicate number of substitutions per nucleotide position. Multiple strains from the same country sharing the same node were collapsed and shown as triangles with shape proportional to branch distances and number of sequences.

Given their relevance in this study, only phylogenies for serotypes CVB1, CVA10, and CVA6 are shown (Figure 1, Figure 2, and Figure 3). The PCR strategy amplified a region that ranged from 1,084 nt (CVB1) to 1,174 nt (CVA6). Notably, most currently available sequences from these serotypes cover only a fragment of the VP1 coding region (either the 3′ or the 5′ part), with typical lengths of ≈300 nt and 400 nt, respectively. In consequence, to attain a global view of the relationships between our sequences and representative isolates circulating worldwide, we performed 2 parallel phylogenetic reconstructions using each part of the coding region.

### CVB1

Nine serotype CVB1 sequences were obtained from 6 onychomadesis case-patients (5 with previous HFMD) and 3 healthy classmates. Eight of the 9 CVB1 sequences were virtually identical (99.9%) and shared 94% nucleotide identity with the relatively divergent 54693 isolate. These CVB1 isolates from Valencia clustered together and were phylogenetically close to isolates circulating in the United States during 2007–2008 (nucleotide identities 96.6%–99.0%) (Figure 1, panel A) and to isolates circulating in Spain in 2008–2009, including those isolates detected in the onychomadesis outbreak reported in A Coruña in 2009 (*15*) (97.7%–99.5%) (Figure 1, panel B).

### CVA10

Serotype CVA10 was detected in 18 children: 12 onychomadesis case-patients (10 with HFMD) and 6 healthy children. Phylogenetic analyses with representative CVA10 sequences are shown in Figure 2. Both 5′ and 3′ partial VP1 analyses (panels A and B, respectively) showed that all but 1 of the CVA10 sequences from the current outbreak clustered with a nucleotide identity of 97%–100%. This CVA10 group showed a close relationship with Slovakian sequences collected in 2007, with a clade that comprises many strains circulating in the People’s Republic of China during 2008–2010 and showed pairwise identities of ≈80% compared with the main cluster of 2008 isolates from Finland that contains the divergent isolate 54602, collected in childcare center 1. Nucleotide identities between isolate 54602 and sequences in the 2008 Finland cluster were 94%–100%.

### CVA6

CVA6 serotype was found in 6 children: 3 from the onychomadesis outbreak reported in fall 2008 (childcare center 9) and 3 from patients with sporadic onychomadesis cases reported in summer 2008. In contrast to the collection of other isolates (except 54636), the samples from the HFMD cluster in childcare center 9 were collected ≈1 month before onychomadesis onset. Phylogenetic relationships of CVA6 isolates (Figure 3) showed that 4 of our isolates formed a cluster with an isolate collected in Great Britain in 2008. These highly similar isolates (identities ≈100%) formed a relatively distant sister cluster to a group that included most CVA6 isolates circulating in the 2008 HFMD outbreak in Finland. In contrast to the results shown above for the CVA10 serotype, these CVA6 isolates from Spain and Finland showed higher nucleotide identities (94%–98%). On the other hand, the divergent CVA6 sequence from isolate 54698 tended to cluster with isolates circulating in Japan, Iceland, and Greece during 2007 and with a divergent isolate that circulated in the 2008 Finland outbreak. Isolate 54657 (a short CVA6 sequence not included in the phylogenetic analyses) was found in a mixed infection with CVB1 in a child with HFMD–onychomadesis (childcare center 3). This partial sequence was identical to sequence 54694 and could likely group within the cluster of Spanish sequences.

### Other Serotypes

Three CVA5 isolates were detected in different children with onychomadesis after HFMD. Isolates 54602 and 54633 were identical and similar to isolate 54678 and to an isolate from China that was circulating in 2008 (data not shown).

Two EV71 sequences, which shared a 99.6% nt identity, were detected in samples from patients with sporadic onychomadesis cases, with and without previous HFMD. Phylogenetic analysis indicated (data not shown) that both EV71 isolates clustered with sequences circulating Europe during 2006–2009.

One CVA16 isolate was detected in a child with sporadic HFMD followed by onychomadesis (isolate 54682); the child was not attending any childcare center. Phylogenetic analysis indicated the isolate grouped with endemic strains that were circulating in China during 2000–2008 (data not shown).

Serotype echovirus 9 (E9) was detected in a dual infection with CVA10 in isolate 54629. Phylogenetic analysis grouped this strain within a cluster of numerous isolates circulating in Spain (2003–2008), Australia (2005–2006), and United Kingdom (2007–2008) (data not shown).

## Discussion

The main EVs detected in the HFMD–onychomadesis outbreak in Valencia in 2008 included HEV-A serotypes that caused HFMD (CVA6 and CVA10) and an HEV-B serotype (CVB1), currently associated with meningitis and myocarditis and detected recently in clusters of severe systemic neonatal illness (*25*) and onychomadesis outbreaks (*14**,**15*).

The other EVs detected in our survey (CVA5, EV71, CVA16, and E9) could be incidental to the outbreak because they were found rarely and, except for CVA16, were identified from fecal samples collected long after HFMD onset (*26*). In fact, if more stringent exclusion criteria had been followed, they would have been excluded from the analysis. For instance, the time from onset of HFMD and onychomadesis symptoms to specimen collection for isolate 54678 (90 and 47 days, respectively) and from onychomadesis onset to specimen collection in isolate 54720 (61 days), although both contained EV71 serotype, was considerably longer than the average from symptom onset to fecal sample collection (44 and 25 days for HFMD and onychomadesis, respectively). This delay could also be the case for E9, which was detected only once in a mixed infection (but detected in 3 excluded samples collected >90 days after HFMD onset).

Phylogenetic analyses found that divergent strains within serotypes CVA6 and CVA10 were isolated in the contemporary HFMD–onychomadesis outbreaks in Valencia and Finland. Phylogenies of CVB1 showed that a virtually identical CVB1 strain was detected in both onychomadesis outbreaks in Valencia and A Coruña (*15*), along with a relatively divergent strain from Valencia. Consequently, no single serotype or strain within serotype can account exclusively for onychomadesis. The same conclusion arises after considering serotyping results from previous HFMD–onychomadesis studies in which CVA6 (*16*), CV6 and CV10 (*7*), and CVB1 (*15*) or CVB1 and CVB2 (*14*) were detected as single infections. On the contrary, we found, although in a low proportion, dual infections of CVA10/CVB1 and CVA6/CVB1 in onychomadesis cases that led us to suggest that a mixed infection of serotypes from 2 different EV species might account for this unexpected and late complication. Two different serotypes could have infected patients either simultaneously (co-infection) or sequentially (superinfection). Constituents of this mixed infection that possibly causes HFMD–onychomadesis would be 1 HEV-A serotype that causes HFMD, CVA10 or CVA6, and 1 HEV-B serotype, CVB1, never found before in clusters of only HFMD. These 3 serotypes co-circulated during spring and fall 2008 in Valencia and accounted for a cumulative 85.5% of all detected infections in our study.

Identifying serotype CVB1 as a cofactor that contributes to onychomadesis in a HFMD context would solve satisfactorily the question of which serotype is responsible for the onychomadesis feature but also would generate new concerns. First, is CVB1 an incident serotype detected in the 3 onychomadesis outbreaks in Spain or a true cofactor? Second, why do typing results among HFMD–onychomadesis studies not agree?

Prevalence of CVB1 in Spain before 2008 was low. Eighteen CVB1 isolates (0.6%) were detected in Spain from 2,814 typed EV isolates, mainly collected from children during 1998–2007 (*27*). The detection of 17 CVB1 isolates collected in 3 distant onychomadesis outbreaks (≈1,000 km from A Coruña to Valencia) during a year (May 2008–April 2009) seems too high to be considered just a chance event.

Differences in specimens and methods may explain the discordant typing results among HFMD–onychomadesis studies, especially in the use of viral culture (*28**,**29*) or different sets of RT-PCR primers (*30**,**31*). In the Saragossa and A Coruña outbreaks, typing was performed after viral culture (*15*). By using the same cell lines, we obtained EV-positive cultures when they were injected with either CVB1 or HEV-A/CVB1 viral extracts, but cultures were EV-negative when they were inoculated with HEV-A strains alone. Similarly, 8 CVA6 RT-PCR–positive specimens could not be cultured in the outbreak in Finland (*16*). Therefore, the method applied in these 2 outbreaks in Spain is highly likely to have missed HEV-A strains, even if present, and selected for, and indeed found, only CVB1 isolates.

A noteworthy inconsistency, if our hypothesis holds, is that CVB1 was not detected in the HFMD–onychomadesis outbreak in Finland. Specimens studied in the 3 Spanish surveys were sampled a long time after HFMD diagnosis, whereas in the 2 surveys from Finland, acute-phase specimens were obtained (*7**,**16*). Typing methods in Finland (*16*) were based on primers specific for CVA6-VP1 or melting point comparison in the 5′ noncoding region, which are not advised for typing at the serotype level. Not surprisingly, only the CVA6 serotype was found. In fact, the cited study could not detect co-circulating CV10. The second publication (*7*) about the same outbreak, in which a different method was used, proved co-circulation of CVA6 and CVA10. In this report, protocols seem suitable for detecting any serotype, but the researchers did not specify the number of fecal samples (and their collection data) that were typed directly from onychomadesis case-patients. Curiously, only supernatants from consecutive cultures that showed 100% cytopathic effect underwent typing. However, in the surveys from A Coruña (*15*) and Valencia, all viral cultures were tested, irrespective of cytopathic effect, and CVB1 was detected in A Coruña (viral cultures were not typed in our survey). The highly stringent conditions used in the Finland survey could have seriously compromised the ability to detect CVB1, even if it was initially present.

Our study combined the use of fecal samples along with 3 distinct strategies for molecular amplification of isolates, thus improving previous strategies used in onychomadesis outbreaks. That all EV-positive samples could be typed and that the variability detected was high clearly supported the robustness of our approach.

Our study has some limitations, however. First, virions in feces correspond to viral shedding from the whole patient and add an eventual possibility of detecting incidental EVs. Moreover, the possibility of detecting incidental serotypes increases with time between HFMD onset and specimen collection as reflected in typing results from excluded samples. Therefore, we recommend limiting sample collection to <2 months after HFMD onset. Second, our hypothesis has no statistical support because a Fisher exact test (applied to a 2-way contingency table) performed with our data failed to detect a significant association between the presence of CVA6/CVB1 or CVA10/CVB1 mixed infections and onychomadesis. However, this lack of significant association does not necessarily invalidate our hypothesis because the quality of the specimens may affect detection of some serotypes and, consequently, the result of the test. For instance, distant serotypes may differ in their persistence pattern in feces (*26*), which could have led to the poor association between mixed infections detected and expected according to our hypothesis. Finally, our studied population was temporally and geographically restricted.

Further research of HFMD–onychomadesis outbreaks will be needed to confirm or negate our hypothesis. Adequate specimens to test the hypothesis would comprise, ideally, nail sampling and serial fecal sampling from time of HFMD diagnosis to ≈2 months after HFMD onset.

The standardization of protocols and techniques in typing is essential for EV surveillance and worldwide comparisons. In this context, we strongly recommend that the complete VP1 gene be sequenced.
